# Supplementary material for: LTA4H extensively associates with mRNAs and lncRNAs indicative of its novel regulatory targets
Source: PeerJ. 2023 Mar 10;11:e14875. doi: 10.7717/peerj.14875 (PMC10010175; doi:10.7717/peerj.14875)
Supplement: File S2 [file peerj-11-14875-s005.zip › Raw data files/Quality_control/FastqcView/LTA4H_IP_1_fastqc/fastqc_report.html]

E743-IP-1-mRNA\_all.fq.clean\_fq\_reverse FastQC Report


FastQC Report

Mon 16 Oct 2017  
E743-IP-1-mRNA\_all.fq.clean\_fq\_reverse

## Summary

- Basic Statistics
- Per base sequence quality
- Per sequence quality scores
- Per base sequence content
- Per base GC content
- Per sequence GC content
- Per base N content
- Sequence Length Distribution
- Sequence Duplication Levels
- Overrepresented sequences
- Kmer Content

## Basic Statistics

| Measure | Value |
| --- | --- |
| Filename | E743-IP-1-mRNA\_all.fq.clean\_fq\_reverse |
| File type | Conventional base calls |
| Encoding | Sanger / Illumina 1.9 |
| Total Sequences | 29540199 |
| Sequence length | 16-147 |
| %GC | 51 |

## Per base sequence quality

## Per sequence quality scores

## Per base sequence content

## Per base GC content

## Per sequence GC content

## Per base N content

## Sequence Length Distribution

## Sequence Duplication Levels

## Overrepresented sequences

| Sequence | Count | Percentage | Possible Source |
| --- | --- | --- | --- |
| TGGTAGTAGCAAATATTCAAACGAGAACTTTGAAGGCCGAAGTGGAGAAG | 300703 | 1.0179450720694196 | No Hit |
| TGGTGGTAGTAGCAAATATTCAAACGAGAACTTTGAAGGCCGAAGTGGAG | 170698 | 0.5778498648570377 | No Hit |
| AGCCTAGGGCGCGGGCCCGGGTGGAGCCGCCGCAGGTGCAGATCTTGGTG | 137193 | 0.4644281509410279 | No Hit |
| AATATTCAAACGAGAACTTTGAAGGCCGAAGTGGAGAAGGGTTCCATGTG | 136651 | 0.46259336303049275 | No Hit |
| CCTATTCTCAAACTTTAAATGGGTAAGAAGCCCGGCTCGCTGGCGTGGAG | 127848 | 0.43279329296325997 | No Hit |
| ACTTTAAATGGGTAAGAAGCCCGGCTCGCTGGCGTGGAGCCGGGCGTGGA | 105589 | 0.3574417355820792 | No Hit |
| GTCCGCCGAGGGCGCACCACCGGCCCGTCTCGCCCGCCGCGCCGGGGAGG | 94578 | 0.32016710517082164 | No Hit |
| TGAAGCCTAGGGCGCGGGCCCGGGTGGAGCCGCCGCAGGTGCAGATCTTG | 88963 | 0.301159108643784 | No Hit |
| CAGCAGGACGGTGGCCATGGAAGTCGGAATCCGCTAAGGAGTGTGTAACA | 87064 | 0.29473058052181705 | No Hit |
| ATCAGACGTGGCGACCCGCTGAATTTAAGCATATTAGTCAGCGGAGGAGA | 84844 | 0.28721539756722697 | No Hit |
| ATTTTGTTGGTTTTCGGAACTGAGGCCATGATTAAGAGGGACGGCCGGGG | 84589 | 0.2863521670927132 | No Hit |
| GGCCCTTGAAAATCCGGGGGAGAGGGTGTAAATCTCGCGCCGGGCCGTAC | 83419 | 0.2823914625625914 | No Hit |
| CCTTGAAAATCCGGGGGAGAGGGTGTAAATCTCGCGCCGGGCCGTACCCA | 82123 | 0.2780042206215334 | No Hit |
| CAGATCAGACGTGGCGACCCGCTGAATTTAAGCATATTAGTCAGCGGAGG | 72444 | 0.24523869998302994 | No Hit |
| TTGGTTTTCGGAACTGAGGCCATGATTAAGAGGGACGGCCGGGGGCATTC | 71585 | 0.24233079811006014 | No Hit |
| AGACAGCAGGACGGTGGCCATGGAAGTCGGAATCCGCTAAGGAGTGTGTA | 70540 | 0.2387932457733274 | No Hit |
| TGGAATGCGAGTGCCTAGTGGGCCACTTTTGGTAAGCAGAACTGGCGCTG | 66337 | 0.22456517642281287 | No Hit |
| TAGTGACGCGCATGAATGGATGAACGAGATTCCCACTGTCCCTACCTACT | 64166 | 0.21721586912803126 | No Hit |
| CTTTAAATGGGTAAGAAGCCCGGCTCGCTGGCGTGGAGCCGGGCGTGGAA | 60669 | 0.20537776336577826 | No Hit |
| GGCGTGGAGCCGGGCGTGGAATGCGAGTGCCTAGTGGGCCACTTTTGGTA | 58693 | 0.19868857349268362 | No Hit |
| TGGCGTGGAGCCGGGCGTGGAATGCGAGTGCCTAGTGGGCCACTTTTGGT | 55444 | 0.18769000168211458 | No Hit |
| GGAGCCCCGCGGACGCTACGCCGCGACGAGTAGGAGGGCCGCTGCGGTGA | 49620 | 0.16797449468773043 | No Hit |
| GCCATGATTAAGAGGGACGGCCGGGGGCATTCGTATTGCGCCGCTAGAGG | 48618 | 0.16458250670552355 | No Hit |
| TGGTTTTCGGAACTGAGGCCATGATTAAGAGGGACGGCCGGGGGCATTCG | 47647 | 0.16129546046727714 | No Hit |
| AGCTGGTTCCCTCCGAAGTTTCCCTCAGGATAGCTGGCGCTCTCGCAGAC | 47461 | 0.1606658100035142 | No Hit |
| TGGCCCTTGAAAATCCGGGGGAGAGGGTGTAAATCTCGCGCCGGGCCGTA | 44951 | 0.15216891396026141 | No Hit |
| AATTAGTGACGCGCATGAATGGATGAACGAGATTCCCACTGTCCCTACCT | 43852 | 0.14844855987598457 | No Hit |
| CGGCGGGAGTAACTATGACTCTCTTAAGGTAGCCAAATGCCTCGTCATCT | 39871 | 0.13497200882092905 | No Hit |
| CTACGGGGTCTGACGCTCAGTGGAACGAAAACTCACGTTAAGGGATTTTG | 39121 | 0.13243309566059455 | No Hit |
| GTGGAGCCGGGCGTGGAATGCGAGTGCCTAGTGGGCCACTTTTGGTAAGC | 36663 | 0.12411223092979164 | No Hit |
| CACGGTGAAGAGACATGAGAGGTGTAGAATAAGTGGGAGGCCCCCGGCGC | 35707 | 0.12087596295475193 | No Hit |
| TATAGGGGCGAAAGACTAATCGAACCATCTAGTAGCTGGTTCCCTCCGAA | 35297 | 0.11948802376043574 | No Hit |
| ACGGTGAAGAGACATGAGAGGTGTAGAATAAGTGGGAGGCCCCCGGCGCC | 33250 | 0.11255848344149612 | No Hit |
| GATCTTGGTGGTAGTAGCAAATATTCAAACGAGAACTTTGAAGGCCGAAG | 33169 | 0.11228428082018 | No Hit |
| CCTAGGGCGCGGGCCCGGGTGGAGCCGCCGCAGGTGCAGATCTTGGTGGT | 32923 | 0.11145151730359026 | No Hit |
| ACCGTCGTAGTTCCGACCATAAACGATGCCGACCGGCGATGCGGCGGCGT | 32115 | 0.10871626152552324 | No Hit |
| GCATTGAATGAATTTAAATCATCTTCTTTGGCAGTAATCCATCTTTGACT | 31998 | 0.10832019107251106 | No Hit |
| AAATCCGGGGGAGAGGGTGTAAATCTCGCGCCGGGCCGTACCCATATCCG | 31865 | 0.10786995713874506 | No Hit |
| TTGGCAGTAATCCATCTTTGACTTAAGGCAATACAAGCATTTGTCAGAGT | 31191 | 0.10558832051199114 | No Hit |
| GGTTTTCGGAACTGAGGCCATGATTAAGAGGGACGGCCGGGGGCATTCGT | 30703 | 0.10393633434900015 | No Hit |
| GCTAGGAATAATGGAATAGGACCGCGGTTCTATTTTGTTGGTTTTCGGAA | 30673 | 0.10383477782258677 | No Hit |
| CAGTGCTCTGAATGTCAAAGTGAAGAAATTCAATGAAGCGCGGGTAAACG | 30517 | 0.10330668388523719 | No Hit |
| CCGCGACGAGTAGGAGGGCCGCTGCGGTGAGCCTTGAAGCCTAGGGCGCG | 30085 | 0.10184426990488453 | No Hit |

## Kmer Content

| Sequence | Count | Obs/Exp Overall | Obs/Exp Max | Max Obs/Exp Position |
| --- | --- | --- | --- | --- |
| ACTTT | 6053145 | 3.0491333 | 7.1566014 | 25-29 |
| TTTTT | 4380250 | 2.8594983 | 10.703882 | 130-134 |
| CTTTG | 5961645 | 2.8492718 | 7.0552387 | 25-29 |
| CGCCG | 8487850 | 2.8288815 | 5.9048967 | 4 |
| TGAAC | 8778520 | 2.7532961 | 9.331817 | 70-74 |
| TTCCC | 4546390 | 2.564927 | 5.5891185 | 7 |
| TTTTC | 4024690 | 2.502625 | 9.332668 | 130-134 |
| TTGAA | 7564665 | 2.4908574 | 6.4981422 | 30-34 |
| TTGGT | 6300680 | 2.4298928 | 5.9801545 | 45-49 |
| GTTTT | 4817655 | 2.4173024 | 6.554484 | 120-124 |
| AACTT | 5698420 | 2.3253145 | 7.5456753 | 25-29 |
| TTTTG | 4609735 | 2.3129764 | 6.0446534 | 2 |
| CTGGC | 6277740 | 2.1965847 | 6.0255456 | 85-89 |
| GAACT | 6984620 | 2.1906571 | 5.9083657 | 25-29 |
| CTTTT | 3486235 | 2.1678042 | 5.2908425 | 70-74 |
| GTTCC | 4746530 | 2.1608093 | 6.449161 | 100-104 |
| TCCCT | 3718640 | 2.097937 | 6.134984 | 8 |
| TATTC | 4064530 | 2.0474138 | 15.506858 | 3 |
| CCCTC | 3747405 | 2.0137773 | 8.722858 | 140-142 |
| GGCGC | 7485770 | 2.0131934 | 5.3841786 | 90-94 |
| GCCGC | 5963195 | 1.9874493 | 5.53827 | 25-29 |
| TGTTG | 5102335 | 1.9677442 | 7.019414 | 5 |
| TGGTT | 5099335 | 1.9665872 | 5.108695 | 8 |
| TGGTG | 6607160 | 1.9584786 | 9.457594 | 1 |
| CCATG | 5297095 | 1.953484 | 7.247202 | 55-59 |
| CCGCC | 4706975 | 1.9441378 | 6.3733068 | 3 |
| GAACA | 7599880 | 1.9309467 | 5.4461093 | 60-64 |
| ATTTT | 3633955 | 1.9217746 | 5.9659195 | 1 |
| GGTGG | 8380495 | 1.9093174 | 7.5660057 | 2 |
| TTTAA | 4443940 | 1.903807 | 5.634913 | 3 |
| CATTT | 3768110 | 1.8980992 | 5.426025 | 105-109 |
| GGTTC | 5128910 | 1.8840708 | 6.065893 | 50-54 |
| GGCCG | 6968240 | 1.8740109 | 5.8497677 | 35-39 |
| ATATT | 4361645 | 1.8685517 | 7.382778 | 2 |
| CCTCC | 3458700 | 1.8586332 | 6.22994 | 120-124 |
| AAATA | 6588845 | 1.8523707 | 5.1132784 | 10-14 |
| TTTGT | 3645535 | 1.8291804 | 5.8682346 | 3 |
| GCCTC | 4207440 | 1.8244443 | 5.1390595 | 120-124 |
| AGCAG | 7494635 | 1.8067019 | 6.701915 | 2 |
| TTCAA | 4331130 | 1.767374 | 7.887279 | 5 |
| CTCTG | 3825450 | 1.741497 | 5.220879 | 120-124 |
| CCTCT | 3084830 | 1.7403618 | 5.5889387 | 80-84 |
| TGAAG | 6842140 | 1.7316315 | 5.319573 | 30-34 |
| TCAAA | 5176265 | 1.7111012 | 5.7576914 | 6 |
| ATTCA | 4141865 | 1.6901419 | 7.840311 | 4 |
| TCTCC | 2936265 | 1.6565462 | 5.513213 | 8 |
| AGCCT | 4430010 | 1.6337172 | 7.4850864 | 1 |
| TGGGT | 5489920 | 1.6273091 | 5.2504463 | 9 |
| CCAAG | 5402765 | 1.6140617 | 5.3081985 | 1 |
| CAGCA | 5401560 | 1.6137017 | 7.7365174 | 1 |
| CATGT | 4159545 | 1.6104436 | 5.923725 | 55-59 |
| CAAAT | 4849640 | 1.6031296 | 5.847685 | 10-14 |
| TTGTT | 3193810 | 1.6025233 | 6.4199066 | 4 |
| ATTCT | 3146945 | 1.5852015 | 8.188396 | 4 |
| GCCCT | 3610690 | 1.5656796 | 7.2565 | 140-142 |
| TGTTT | 3119010 | 1.5649917 | 5.6147556 | 120-124 |
| GAAAG | 7530710 | 1.5439433 | 6.4131284 | 140-142 |
| GCAGG | 6681045 | 1.5281037 | 6.1353974 | 3 |
| GCTGG | 5319075 | 1.5018017 | 5.0517364 | 2 |
| TGGTA | 4787480 | 1.4956795 | 14.311737 | 1 |
| CAAAC | 4742365 | 1.4932297 | 6.182637 | 7 |
| AATAT | 4256750 | 1.4772869 | 5.804504 | 1 |
| TCAGT | 3739955 | 1.4479916 | 6.7127004 | 80-84 |
| CTTTA | 2851605 | 1.4364307 | 6.412523 | 2 |
| ACTCT | 2961310 | 1.420861 | 6.6326284 | 120-124 |
| GCAAA | 5540055 | 1.4075948 | 11.567525 | 9 |
| TTGCC | 3081260 | 1.4027121 | 6.234109 | 125-129 |
| TTTCA | 2770965 | 1.3958101 | 5.9024777 | 130-134 |
| TTCTC | 2356135 | 1.39552 | 8.991183 | 5 |
| AACGA | 5468730 | 1.3894727 | 5.396487 | 20-24 |
| TAGTA | 4178860 | 1.3759955 | 14.849803 | 4 |
| CGCCC | 3320420 | 1.3714442 | 5.1468434 | 120-124 |
| GCAGT | 4572360 | 1.3606439 | 5.1524982 | 65-69 |
| TTAAT | 3165275 | 1.3560206 | 5.406612 | 135-139 |
| ATGCC | 3656925 | 1.3486158 | 6.560928 | 140-142 |
| CCTTG | 2945405 | 1.3408656 | 5.6986127 | 4 |
| GTCAG | 4504210 | 1.3403637 | 5.2543063 | 80-84 |
| CGAAA | 5240835 | 1.3315701 | 7.3868737 | 140-142 |
| GTAGC | 4447525 | 1.3234955 | 13.309832 | 6 |
| ACCCC | 3031125 | 1.3195217 | 5.2052817 | 115-119 |
| TCATT | 2618870 | 1.3191959 | 6.3536744 | 125-129 |
| ACGAG | 5197635 | 1.2529731 | 5.056605 | 20-24 |
| CCCTT | 2191350 | 1.2362891 | 7.086468 | 3 |
| GTGGT | 4145650 | 1.2288438 | 9.19089 | 3 |
| ATCAA | 3644245 | 1.2046663 | 5.1135178 | 130-134 |
| CTATT | 2381280 | 1.1995153 | 8.088854 | 2 |
| AGTAG | 4738765 | 1.1993024 | 11.751245 | 5 |
| CTCAT | 2482290 | 1.1910235 | 7.0442185 | 135-139 |
| TTCAT | 2354165 | 1.1858568 | 5.4748116 | 125-129 |
| CAGAC | 3968105 | 1.1854608 | 5.1011024 | 3 |
| TCCGC | 2721000 | 1.1798893 | 5.511888 | 2 |
| GTAGT | 3767175 | 1.1769211 | 14.068895 | 3 |
| CGCTC | 2709520 | 1.1749113 | 5.894791 | 135-139 |
| AGCAA | 4556955 | 1.1578127 | 11.503963 | 8 |
| GCTCA | 3102820 | 1.1442705 | 5.6028423 | 135-139 |
| CCTAG | 3082790 | 1.1368839 | 6.6338344 | 3 |
| GCCTA | 3064215 | 1.1300336 | 7.032519 | 2 |
| TCTCA | 2345735 | 1.1255033 | 7.5809517 | 6 |
| CGATG | 3746610 | 1.1149172 | 5.7161565 | 125-129 |
| CGGTC | 3141245 | 1.0991234 | 5.348396 | 85-89 |
| AGGGC | 4781925 | 1.0937327 | 6.098855 | 9 |
| TAGCA | 3480770 | 1.091709 | 13.908138 | 7 |
| CCGAC | 2932595 | 1.030141 | 5.6739717 | 130-134 |
| TATAG | 2978240 | 0.9806609 | 5.596316 | 140-142 |
| CCGAT | 2651055 | 0.9776668 | 7.5822525 | 135-139 |
| TGCCG | 2764085 | 0.96715486 | 5.302031 | 140-142 |
| GGTAG | 3877220 | 0.9310145 | 10.807143 | 2 |
| TGCCC | 2127660 | 0.92260313 | 7.405896 | 140-142 |
| TCGGT | 2501175 | 0.9187899 | 5.9751577 | 85-89 |
| TCCGT | 1932645 | 0.87981695 | 6.8229637 | 135-139 |
| CTCAA | 2253675 | 0.87597364 | 6.183111 | 7 |
| CCTAT | 1815825 | 0.871248 | 7.774365 | 1 |
| CATTA | 2110365 | 0.8611619 | 5.0697007 | 125-129 |
| TCAAG | 2569910 | 0.8060268 | 5.1188164 | 140-142 |
| CTAGG | 2570875 | 0.76504153 | 6.6043215 | 4 |
| ATCGA | 1478590 | 0.46374512 | 6.125247 | 140-142 |
| GATCG | 1450650 | 0.43168473 | 5.9065614 | 140-142 |

Produced by FastQC (version 0.9.5)
